# The protein tyrosine phosphatase PTPN22 negatively regulates presentation of immune complex derived antigens

**DOI:** 10.1038/s41598-018-31179-x

**Published:** 2018-08-23

**Authors:** Fiona Clarke, Harriet A. Purvis, Cristina Sanchez-Blanco, Enrique Gutiérrez-Martinez, Georgina H. Cornish, Rose Zamoyska, Pierre Guermonprez, Andrew P. Cope

**Affiliations:** 1 Centre for Inflammation Biology and Cancer Immunology, School of Immunology and Microbial Sciences, Faculty of Life Sciences and Medicine, King’s College London, London, SE1 1UL United Kingdom; 2 Institute of Immunology and Infection Research, Centre for Immunity, Infection and Evolution, University of Edinburgh, Edinburgh, EH9 3FL United Kingdom

## Abstract

A C1858T single nucleotide polymorphism within *PTPN22* (which encodes PTPN22^R620W^) is associated with an enhanced susceptibility to multiple autoimmune diseases including type 1 diabetes and rheumatoid arthritis. Many of the associated autoimmune diseases have an autoantibody component to their pathology. Fc receptors (FcRs) recognise autoantibodies when they bind to autoantigens and form immune complexes. After immune complex binding and receptor crosslinking, FcRs signal via Src and Syk family kinases, leading to antigen uptake, presentation and cytokine secretion. *Ptpn22* encodes a protein tyrosine phosphatase that negatively regulates Src and Syk family kinases proximal to immunoreceptor signalling cascades. We therefore hypothesised that PTPN22 regulates immune complex stimulated FcR responses in dendritic cells (DCs). Bone marrow derived DCs (BMDCs) from wild type (WT) or *Ptpn22*^*−/−*^ mice were pulsed with ovalbumin:anti-ovalbumin immune complexes (ova ICs). Co-culture with WT OT-II T cells revealed that ova IC pulsed *Ptpn22*^*−/−*^ BMDCs have an enhanced capability to induce T cell proliferation. This was associated with an increased capability of *Ptpn22*^*−/−*^ BMDCs to present immune complex derived antigens and to form ova IC dependent DC-T cell conjugates. These findings highlight PTPN22 as a regulator of FcR mediated responses and provide a link between the association of PTPN22^R620W^ with autoantibody associated autoimmune diseases.

## Introduction

The C1858T single nucleotide polymorphism in the human protein tyrosine phosphatase non-receptor type 22 (*PTPN22*) gene encodes an R620W missense mutation in PTPN22 and is associated with an increased susceptibility to multiple autoimmune diseases including rheumatoid arthritis (RA)^1–4^, systemic lupus erythematosus (SLE)^5,6^ and type 1 diabetes (T1D)^7–9^. PTPN22 (also known as PEP in the mouse) was first described as a negative regulator of Src and Syk family kinases downstream of the T cell receptor (TCR). In keeping with this function, *Ptpn22*^*−/−*^ mice were subsequently reported to display enhanced TCR signalling that results in expansion of CD4^+^ effector T cells^10^. PTPN22 also regulates signalling downstream of additional receptors in various cell subsets, including the B cell receptor^11^, the αLβ2 integrin LFA-1^12^, Toll-like receptors (TLRs)^13^ and dectin-1^14^. Furthermore, PTPN22 functions to alter Src and Syk family kinase independent signalling events by regulating TRAF ubiquitination^15^. The R620W mutation is located in the P1 domain of PTPN22, which causes diminshed binding to the inhibitory tyrosine kinase Csk^16,17^. How the expression of PTPN22^R620W^ affects the functions of different immune cells is not straight forward. Both gain- and loss-of-phosphatase function effects have been observed when investigating different signalling pathways in different cell types^11,17–20^.

Autoantibodies have long been implicated in the aetiology of autoimmune diseases including RA, type 1 diabetes, Graves’ disease and SLE; diseases for which *PTPN22*^*R620W*^ is also a susceptibility risk allele^21^. Autoantibodies bind to self-antigens forming immune complexes which are recognised by Fc receptors (FcRs), thus inducing FcR mediated antigen uptake and cell activation. FcRs are expressed on the surface of most innate immune cells and are members of the immunoglobulin superfamily of receptors. FcRs recognise the Fc region of immunoglobulins, with FcγRs specifically recognising the Fc regions of IgGs. Mice express four cell surface FcγRs: FcγRI, IIb, III and IV. FcγRI, III and IV are activatory receptors, whereas FcγRIIb is inhibitory^22^. Most innate immune cells express both activatory and inhibitory FcγRs, allowing for the modulation of downstream signalling. Activatory FcγR crosslinking induces Src family kinase activation, which in turn phosphorylates two tyrosine residues in the immunoreceptor tyrosine-based activation motif (ITAM), located in the associated common γ chain. Syk is then recruited via its tandem SH2 domains to the phosphorylated tyrosines. This initiates downstream signalling involving ERK, p38 and JNK, activating a range of cellular processes including DC maturation and cytokine production^23^. For the inhibitory receptor FcγRIIb, phosphatases such as SH2-domain-containing protein tyrosine phosphatase 1 (SHP1) and SH2-domain-containing inositol polyphosphate 5′ phosphatase (SHIP1) are recruited to the immunoreceptor tyrosine-based inhibition motif (ITIM), located in the cytoplasmic tail of the receptor. Co-ligation of an activatory FcγR with an inhibitory FcγR reduces activatory signalling by dephosphorylation of signalling intermediates. Therefore, the cellular response to FcR signalling is dependent on the balance between the positive and negative signals. The necessity for appropriate regulation of FcγR signalling is demonstrated by the presence of polymorphisms in human *FCGR* genes which are linked to autoimmune diseases such as SLE, RA and multiple sclerosis^24^. Furthermore, mice lacking expression of the activatory FcγRs are resistant to a variety of autoimmune disease models such as collagen-induced arthritis^25^, but are susceptible to infections including *Mycobacterium tuberculosis*^26^.

ITAM signalling downstream of FcγRs in BMDCs is required for optimal T cell responses. This was demonstrated using NOTAM BMDCs, which are unable to signal through ITAMs, but have normal FcγR cell surface expression^27^. NOTAM BMDCs show normal ovalbumin:anti-ovalbumin immune complex (ova IC) binding, but reduced uptake and degradation. As a result, NOTAM BMDCs are unable to present ova IC derived antigens on MHCI and MHCII (despite being capable of stimulating T cells when pulsed with ova peptide or protein)^28^. ITAM signalling was also demonstrated to be important using DAP12 and FcRγ doubly deficient mice, where BMDCs pulsed with ova coated beads were unable to cause OT-II T cell proliferation^29^.

Given the link between *PTPN22*^*R620W*^ and autoantibody associated autoimmune diseases, and the regulation of FcRs by Src and Syk family kinases, we set out to investigate if PTPN22 regulates FcγR dependent immune complex uptake and activation in DCs and whether this can alter T cell effector responses.

## Results

### Immune complex pulsed *Ptpn22*^*−/−*^ BMDCs cause enhanced T cell proliferation

**Figure 1:**
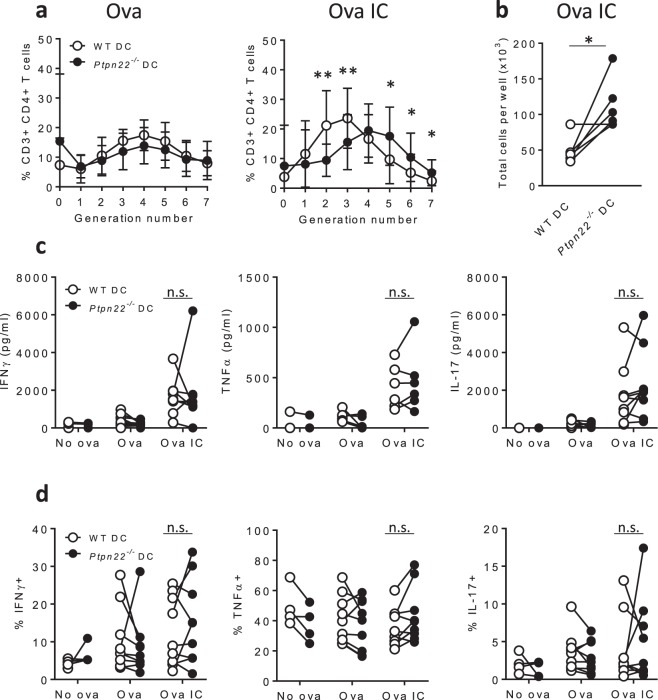
*Ptpn22*^*−/−*^ BMDCs enhance immune complex induced T cell proliferation. (**a**–**d**) WT or *Ptpn22*^*−/−*^ BMDCs were pulsed overnight in the presence of ova or ova IC prior to co-culture with CTV labelled WT CD4^+^ OT-II T cells for 6 days. (**a**) Proportions of WT OT-II T cells in each generation of cell division after 6 days of co-culture with ova (left) and ova IC (right) pulsed WT (white) or *Ptpn22*^*−/−*^ (black) BMDCs (gated on live, CD3^+^, CD4^+^ singlets). n = 10 ± s.d.; **p < 0.01, *p < 0.05 using a Wilcoxon matched-pairs signed ranks test. (**b**) The number of cells per well were counted using trypan blue after 6 days of co-culture with ova IC pulsed WT (white) or *Ptpn22*^*−/−*^ (black) BMDCs. n = 6; *p < 0.05 using a paired t-test. (**c**) At day 6 of co-culture cell-free supernatants were assessed for IFNγ (left), TNFα (middle) and IL-17 (right) by immunoassay. n = 3–10. (**d**) At day 6 T cells co-cultured with ova or ova IC pulsed WT (white) or *Ptpn22*^*−/−*^(black) BMDCs were restimulated for 6 hours with PMA and ionomycin in the presence of monensin and intracellular expression of IFNγ (left), TNFα (middle) and IL-17 (right) was determined in live, CD3^+^, CD4^+^ singlets by flow cytometry. n = 4–9. Connecting lines indicate WT or *Ptpn22*^*−/−*^ BMDCs co-cultured with the same WT OT-II T cells. n.s. = not significant using a 2-way ANOVA.

In view of its known substrates, we hypothesised that PTPN22 should negatively regulate FcγR dependent immune responses. To determine whether PTPN22 modulates the capability of DCs to present immune complex derived peptides and in turn activate T cells, we carried out *in vitro* co-culture assays. Wild type (WT) and *Ptpn22*^*−/−*^ BMDCs were pulsed with ovalbumin (ova) and ova immune complexes (ICs) and co-cultured with ova specific WT CD4^+^ OT-II T cells. After 6 days, T cell proliferation was assessed by CellTrace Violet (CTV) dilution. We have previously shown that ova and ova_323–339_ peptide pulsed WT and *Ptpn22*^*−/−*^ BMDCs induce comparable WT CD4^+^ OT-II T cell proliferation^30^. Firstly, we confirmed published data^31^ demonstrating that ova IC pulsed DCs are potent activators of T cells when compared to non-complexed ova antigen, as seen by enhanced T cell proliferation in response to ova IC pulsed BMDCs (Fig. 1a, Supplementary Fig. S1). We next compared the capability of ova IC pulsed WT or *Ptpn22*^*−/−*^ BMDCs to induce T cell proliferation. Interestingly, we observed that ova IC pulsed *Ptpn22*^*−/−*^ BMDCs induced enhanced T cell proliferation in comparison to WT BMDCs (Fig. 1a, Supplementary Fig. S1). This was accompanied by an increase in total cell numbers in the co-culture wells on day 6 (Fig. 1b). Enhanced T cell proliferation induced by ova IC pulsed *Ptpn22*^*−/−*^ BMDCs was not explained by differences in T cell viability (Supplementary Fig. S1). Although T cells co-cultured with ova IC pulsed BMDCs secreted enhanced levels of IFNγ, TNFα and IL-17 (Fig. 1c), no consistent *Ptpn22* dependent changes in expression of these cytokines were observed (Fig. 1c,d).

We also measured T cell proliferation and cytokine expression following 1 and 3 days of co-culture, to try to understand at what time point BMDC expression of PTPN22 regulated T cell proliferation. After 1 day of co-culture, CD25 and CD69 expression was enhanced on T cells co-cultured with *Ptpn22*^*−/−*^ BMDCs pulsed with ova ICs (Supplementary Fig. S1). This was also accompanied by augmented IL-2 secretion (Supplementary Fig. S1). Finally, the total number of T cells per well was calculated after 3 days of co-culture, and this was found to be highest when T cells were co-cultured with *Ptpn22*^*−/−*^ BMDCs pulsed with ova ICs (Supplementary Fig. S1).

Together, these data demonstrate that PTPN22 negatively regulates the capability of BMDCs to mediate immune complex dependent T cell activation.

### PTPN22 is dispensable for FcγR induced BMDC maturation

**Figure 2:**
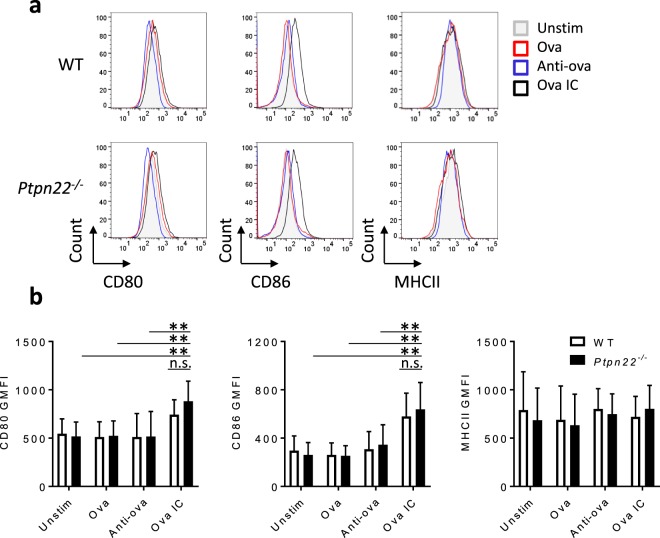
PTPN22 does not regulate immune complex induced maturation of BMDCs. (**a,b**) WT or *Ptpn22*^*−/−*^ BMDCs were stimulated in the presence of ova, anti-ova or ova IC. After 24 hours, BMDCs were harvested and surface stained for CD80, CD86 and MHCII. (**a**) Representative flow cytometry plots showing cell surface expression of CD80, CD86 and MHCII on WT (top) and *Ptpn22*^*−/−*^ (bottom) BMDCs after 24 hour stimulation with ova (red), anti-ova (blue), ova IC (black), or left unstimulated (grey). (**b**) Pooled data (geometric mean fluorescence intensity, GMFI, gated on live, CD11c^+^ singlets) are shown for WT (white) and *Ptpn22*^*−/−*^ (black) BMDC CD80 (left), CD86 (middle) and MHCII (right) expression. n = 6–8 + s.d.; ***p < 0.005, **p < 0.01 using a 2-way ANOVA with Tukey’s multiple comparisons test. All WT v *Ptpn22*^*−/−*^ comparisons were non-significant (n.s.). Bars on graphs only show comparisons of ova IC v unstim/ova/anti-ova for *Ptpn22*^*−/−*^ BMDCs (for simplicity).

We next sought to understand how PTPN22 might regulate the capability of immune complex pulsed BMDCs to induce T cell proliferation. FcγR stimulation induces the maturation of BMDCs, enhancing the cell surface expression of MHCII and co-stimulatory molecules including CD80, CD86 and ICAM-1; molecules capable of modulating T cell activation^32^. We therefore compared the capability of WT and *Ptpn22*^*−/−*^ BMDCs to express co-stimulatory molecules in response to ova ICs. WT and *Ptpn22*^*−/−*^ BMDCs were cultured in the presence of ova ICs (or ova and anti-ova alone) for 24 hours, and upregulation of MHCII and co-stimulatory molecules were assessed. Ova IC stimulation caused a subtle increase in cell surface expression of CD80 and CD86 (Fig. 2a,b), and expression was similar between WT and *Ptpn22*^*−/−*^ BMDCs. This is despite the fact that Syk is required for upregulation of CD40 and CD86 on BMDCs after FcγR crosslinking^33^. As an additional control, WT and *Ptpn22*^*−/−*^ BMDCs were cultured in the presence of ova pre-incubated with rabbit IgG, and ova with heat aggregated rabbit IgG. Neither conditions induced a change in the expression of CD80, CD86 or CD54; once again, these were only upregulated on BMDCs cultured in the presence of ova ICs (Supplementary Fig. S2).

Immune complex stimulation also induces the secretion of T cell polarising cytokines by BMDCs^34,35^. Indeed, we observed that while BMDCs pulsed with ova ICs secreted significantly more IL-6, TNFα and IL-12/23p40 compared to BMDCs incubated with ova alone, both WT and *Ptpn22*^*−/−*^ BMDCs secreted similar levels of these cytokines in response to 24 hours of immune complex stimulation (Supplementary Fig. S3). To further investigate a potential role of PTPN22 in regulating immune complex induced cytokine secretion by BMDCs, we also carried out time course experiments. Secretion of cytokines was measured after 3, 6, 16 and 24 hours of immune complex stimulation. PTPN22 was found to be dispensable for cytokine secretion at all time points (Supplementary Fig. S3).

Ova ICs bind to all FcγRs on BMDCs, so we hypothesised that, instead, selective FcγR binding might uncover differences in cytokine secretion. Therefore, IgG1 immune complexes, which preferentially bind to FcγRIIb and FcγRIII were tested, but these also resulted in similar IL-6 and TNFα secretion from WT and *Ptpn22*^*−/−*^ BMDCs (Supplementary Fig. S3). Together these data indicate that augmented T cell proliferation induced by immune complex stimulated *Ptpn22*^*−/−*^ BMDCs cannot be explained by alterations in BMDC maturation, including upregulation of co-stimulatory molecules and cytokine secretion.

### Immune complex binding, internalisation and degradation occur independently of PTPN22

**Figure 3:**
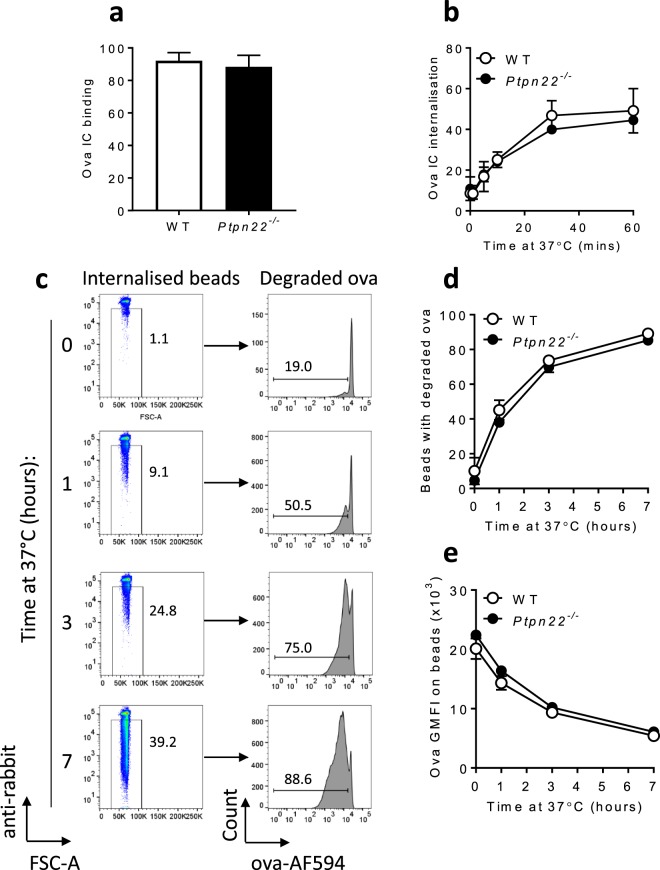
PTPN22 is dispensable for FcγR dependent immune complex binding, uptake and processing. (**a**) WT (white) or *Ptpn22*^*−/−*^ (black) BMDCs were incubated with ova-AF488 ICs for 1 hour on ice. Cell surface ova-AF488 IC binding was determined by flow cytometry, gating on live, CD11c^+^, anti-rabbit^+^ singlets. n = 3–5 + s.d. (**b**) Combined ova-AF488 IC internalisation by WT (white) and *Ptpn22*^*−/−*^ (black) BMDCs was determined by flow cytometry, gating on live, CD11c^+^, anti-rabbit^-^ singlets. n = 3–5 ± s.d. (**c**) Representative flow cytometry plots showing ova-AF594:anti-ova coated bead internalisation and ova degradation by WT BMDCs over 0–7 hours at 37 °C. (**d**) Proportion of beads internalised by WT (white) and *Ptpn22*^*−/−*^ (black) BMDCs that have degraded ova-AF594 on them (gated on single, anti-rabbit^-^ beads). n = 3 ± s.d. (technical repeats, representative of 8 independent experiments). (**e**) Ova-AF594 geometric mean fluorescence intensity (GMFI) of beads internalised by WT (white) and *Ptpn22*^*−/−*^ (black) BMDCs (gated on single, anti-rabbit^-^ beads). n = 3 ± s.d. (technical repeats, representative of 8 independent experiments).

FcγRs are phagocytic receptors that mediate antigen uptake and delivery of antigen into degradation and MHC presentation pathways, that ultimately lead to T cell activation^35^. We therefore sought to assess if PTPN22 regulated IC induced T cell proliferation by modulating the capability of BMDCs to internalise and process antigens. Firstly, we demonstrated that ova IC uptake was indeed FcγR dependent by blocking uptake of fluorescent ova ICs (ova-AF488 ICs) with antibodies against FcγRI, FcγRII/III and FcγRIV (Supplementary Fig. S4). We next replicated findings reported previously^36,37^ that FcγR mediated uptake of immune complexes is dependent on Src and Syk family kinases, known targets of PTPN22. Indeed, pre-incubation of WT BMDCs with small molecular inhibitors of Src and Syk family kinases resulted in a significant reduction in ova-AF488 IC uptake (Supplementary Fig. S4). Having confirmed the dependence of Src and Syk family kinases in mediating IC uptake we next assessed if PTPN22 regulated this process. Despite the requirement for Src and Syk family kinases in internalisation of immune complexes, we found that PTPN22 was dispensable for FcγR dependent binding and uptake of ova-AF488 ICs (Fig. 3a,b).

Following antigen uptake protein antigens are lysosomally degraded into peptides and loaded on to MHCII molecules to form peptide-MHCII complexes. Changes in antigen degradation have the potential to alter the concentration or rate at which antigen is available to be loaded on to MHCII molecules. PTPN22 is required for TRAF3 ubiquitination, ultimately leading to the production of type I interferons^13^, and FcγR ubiquitination has been shown to facilitate internalisation and degradation of immune complexes^38^. Therefore, we next explored the possibility that PTPN22 might regulate the capability of BMDCs to mediate antigen degradation. To address this, WT and *Ptpn22*^*−/−*^ BMDCs were incubated with ova-AF594:anti-ova coated polystyrene beads. Over time the intensity of the ova-AF594 signal is lost, indicative of antigen degradation^30^. However, both the amount of degradation and the proportion of beads with degraded ova were similar in antigen loaded WT and *Ptpn22*^*−/−*^ BMDCs, indicating that ova-AF594:anti-ova degradation also occurs independently of PTPN22 (Fig. 3c–e). Collectively, these data demonstrate that PTPN22 is not required for FcγR dependent immune complex binding, uptake and degradation, showing that these processes are unlikely to be responsible for the *Ptpn22* mediated differences observed in immune complex induced T cell proliferation.

### PTPN22 negatively regulates immune complex derived antigen presentation and DC-T cell conjugate formation

**Figure 4:**
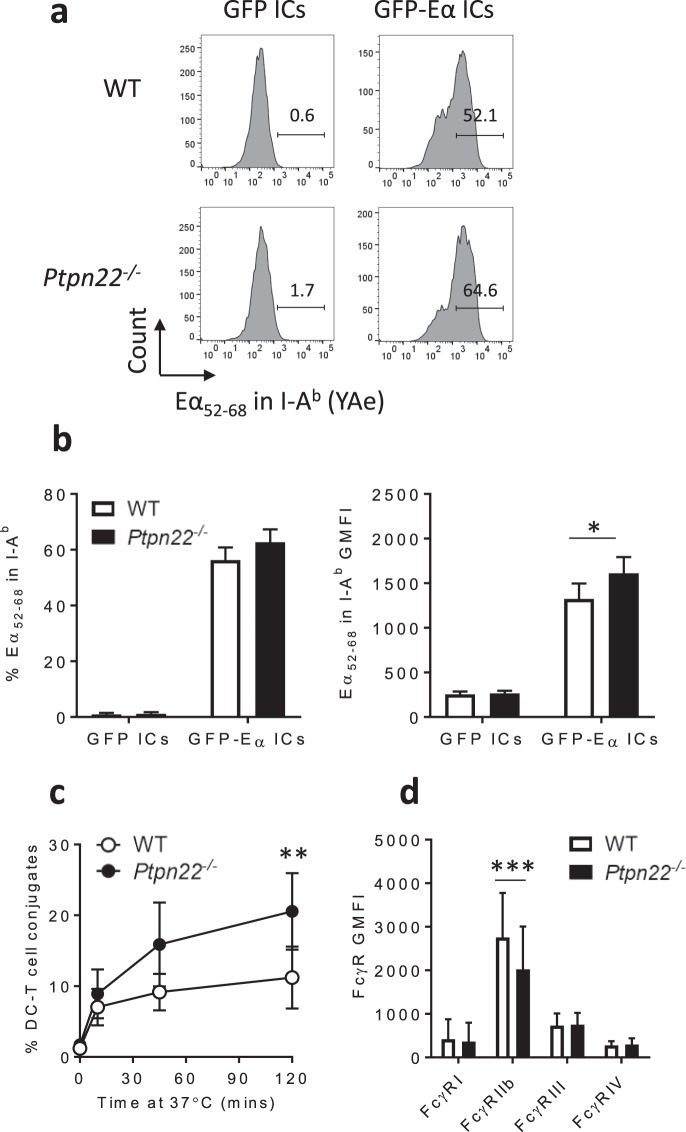
*Ptpn22*^*−/−*^ BMDCs present more immune complex derived antigens and form more conjugates with T cells. (**a**) Representative flow cytometry plots of Eα_52–68_ surface expression in I-A^b^ by WT (top) and *Ptpn22*^*−/−*^ (bottom) BMDCs after 18 hour incubation with GFP:anti-GFP ICs (left) and GFP-Eα:anti-GFP ICs (right). (**b**) Combined proportion (left) and geometric mean fluorescence intensity (GMFI, right) of Eα_52–68_ surface expression in I-A^b^ by WT (white) and *Ptpn22*^*−/−*^ (black) BMDCs after 18 hour incubation with GFP:anti-GFP ICs and GFP-Eα:anti-GFP ICs (gated on live, CD11c^+^ singlets). n = 3 + s.d.; *p < 0.05 using a 2-way ANOVA with Sidak’s multiple comparisons test. (**c**) Proportion of WT OT-II T cells in conjugates with ova IC pulsed WT (white) and *Ptpn22*^*−/−*^ (black) BMDCs over time. Conjugates were identified as CTV^+^ CTFR^+^ events (%, gated on CTV^+^ total T cells). n = 4 ± s.d.; **p < 0.01 using a 2-way ANOVA with Sidak’s multiple comparisons test. (**d**) Cell surface FcγR expression on WT (white) and *Ptpn22*^*−/−*^ (black) BMDCs (gated on live, CD11c^+^ singlets). n = 16 + s.d.; ***p < 0.005 using a 2-way ANOVA with Sidak’s multiple comparisons test.

As FcγR dependent immune complex binding, uptake and processing was found to occur normally in the absence of PTPN22, we set out to determine whether PTPN22 was required for loading of immune complex derived peptide antigens on to MHCII. WT and *Ptpn22*^*−/−*^ BMDCs were incubated with GFP-Eα:anti-GFP immune complexes for 5 or 18 hours, and an antibody (YAe) specific for I-A^b^ restricted presentation of an Eα peptide (Eα_52–68_) was used to determine cell surface presentation of immune complex derived antigens. After 5 hours of incubation, similar proportions of WT and *Ptpn22*^*−/−*^ BMDCs expressed Eα_52–68_ peptide-MHCII complexes at the cell surface (Supplementary Fig. S5). However, when BMDCs were incubated with GFP-Eα:anti-GFP immune complexes for 18 hours (the point at which ova IC pulsed BMDCs are harvested for co-culture assays), although similar proportions of WT and *Ptpn22*^*−/−*^ BMDCs expressed Eα_52–68_ peptide-MHCII complexes at the cell surface (Fig. 4a, b), we observed that *Ptpn22*^*−/−*^ BMDCs expressed higher levels of Eα_52–68_ peptide (as measured by increased geometric mean fluorescence intensity, GMFI, Fig. 4b). Furthermore, the increase in Eα_52–68_ peptide on the cell surface of *Ptpn22*^*−/−*^ BMDCs was not due to differences in MHCII cell surface expression between WT and *Ptpn22*^*−/−*^ BMDCs (Supplementary Fig. S5). Likewise, in agreement with our data showing no role of PTPN22 in IC uptake (Fig. 3b), uptake of the GFP-Eα:anti-GFP immune complexes was comparable in the presence or absence of PTPN22 (Supplementary Fig. S5). We conclude that enhanced expression of peptide-MHCII on immune complex pulsed *Ptpn22*^*−/−*^ BMDCs could, at least in part, be responsible for the increased T cell proliferation observed in Fig. 1. The effect of PTPN22 on Eα_52–68_ peptide-MHCII complex expression was only observed at the later 18 hour time point, indicating that PTPN22 may regulate the rate of peptide-MHCII complex trafficking and/or retention at the cell surface, rather than initial complex formation.

We hypothesised that if *Ptpn22*^*−/−*^ BMDCs were capable of enhanced peptide-MHCII presentation then this would result in enhanced formation of DC-T cell conjugates, which could potentially lead to greater T cell proliferation. To test the requirement for PTPN22 in the formation of DC-T cell conjugates, WT and *Ptpn22*^*−/−*^ BMDCs were pulsed overnight with ova or ova ICs, stained with CellTrace Far Red (CTFR) and incubated with CellTrace Violet (CTV) stained WT OT-II CD4^+^ T cells. We observed that unpulsed and ova pulsed BMDCs formed only low frequencies of CTV^+^ CTFR^+^ conjugates (Supplementary Fig. S6), whereas ova IC pulsed BMDCs formed more conjugates, which increased over time (Fig. 4c). Interestingly, in agreement with our hypothesis, we observed that ova IC pulsed *Ptpn22*^*−/−*^ BMDCs had a significantly enhanced capability to form conjugates with WT OT-II T cells than WT BMDCs (Fig. 4c). PTPN22 does not regulate DC-T cell conjugate formation in all contexts, as we have previously found that conjugate formation of ova_323–339_ peptide pulsed, LPS matured WT and *Ptpn22*^*−/−*^ BMDCs is similar^30^. Together the enhanced capability of *Ptpn22*^*−/−*^ BMDCs to present immune complex derived antigens and form T cell conjugates, could explain the enhanced T cell proliferation induced by immune complex stimulated *Ptpn22*^*−/−*^ BMDCs. How PTPN22 is regulating BMDC enhanced immune complex derived antigen presentation and conjugate formation is unclear. Nonetheless, we observed that immune complex induced DC-T cell conjugate formation was dependent on Src and Syk family kinases (Supplementary Fig. S6), indicating that PTPN22 may be regulating this process via its ability to dephosphorylate these kinases. Finally, we observed altered FcγR expression on the surface of *Ptpn22*^*−/−*^ BMDCs prior to FcγR activation; specifically, reduced expression of the inhibitory FcγRIIb (Fig. 4d, Supplementary Fig. S7). Reduced expression of the inhibitory receptor may alter the activatory/inhibitory (A/I) ratio, thus potentially reducing the negative regulation downstream of activatory FcγRs. These data suggest that PTPN22 acts to regulate FcγR dependent immune responses, reducing the capability of DCs to activate T cells.

## Discussion

Self-antigen immune complexes are a prominent feature of multiple autoimmune diseases including RA, T1D, Graves’ disease and SLE, diseases also associated with *PTPN22*^*R620W*^. FcRs mediate antigen:antibody immune complex internalisation and subsequent immune complexed antigen processing and presentation, leading to the activation of an adaptive T cell immune response. Here we aimed to investigate whether PTPN22 regulates FcγR dependent immune responses. We report that immune complex pulsed *Ptpn22*^*−/−*^ BMDCs are more effective at promoting CD4^+^ T cell proliferation and that this is associated with both enhanced presentation of immune complex derived antigens at the cell surface and DC-T cell conjugate formation. Together, these data demonstrate for the first time that PTPN22 negatively regulates FcγR dependent T cell activation by BMDCs, and suggests that perturbations to PTPN22 regulates DC/T cell activation in response to immune complexes.

Previous investigations have reported conflicting results regarding the role of PTPN22 in FcγR independent internalisation and presentation of ovalbumin by BMDCs^19,39^. However, our own investigations indicate that PTPN22 is dispensable for non-specific antigen uptake and processing^30^. Here, our novel findings suggest that FcγR dependent uptake of ova ICs by *Ptpn22*^*−/−*^ BMDCs results in enhanced T cell activation. Interestingly, we observed that rather than regulating the amount of ova IC internalisation, PTPN22 appears to specifically regulate MHCII restricted antigen presentation by BMDCs and DC-T cell conjugate formation. One explanation for this observation may lie in PTPN22 regulating FcγR dependent antigen compartmentalisation; whereby inflammatory signals promote loading of antigens on to MHCII^40^. In this context, the absence of PTPN22 may confer a subtle but functionally important enhancement in FcγR dependent inflammatory signals that target immune complexed antigen for MHCII presentation. This increase could potentially be due to PTPN22 mediated changes to the balance of inhibitory FcγRIIb and activatory FcγRIII expression on the DC surface (Fig. 4d). PTPN22 has also been demonstrated to negatively regulate the phagocytic fungal pathogen receptor dectin-1, although PTPN22 was found to have no effect on dectin-1 dependent *C. albicans* phagocytosis^14^. However, data presented here suggest that PTPN22 may regulate MHCII presentation of *C. albicans* antigens and subsequent *C. albicans* restricted T cell activation. Together, our data indicate a role for PTPN22 in regulating FcγR dependent MHCII loading, which has the potential to enhance immune complexed self-peptide presentation, increasing subsequent T cell activation.

The role of PTPN22 in the regulation of FcγR signalling has been previously addressed in the context of neutrophils, where PTPN22 is required for optimal immune complex induced ROS production, adhesion and degranulation^41^. In contrast, we observed no differences in FcγR induced BMDC co-stimulatory molecule expression or cytokine secretion, but a more specific role of PTPN22 in the negative regulation of immune complexed antigen presentation by BMDCs. This indicates that the requirement for PTPN22 in the context of FcγR signalling may differ depending on the cell type. Neutrophils are also capable of mediating MHCII antigen presentation^42^, and so it would be interesting to assess if in neutrophils, where PTPN22 has been demonstrated to positively regulate multiple effector functions, FcγR mediated antigen presentation is positively or negatively regulated by PTPN22.

FcγRs internalise immune complexes via receptor mediated endocytosis, a process which utilises Src and Syk family kinases^43^. Syk is essential for FcγR mediated uptake in macrophages^44^, neutrophils^44^ and dendritic cells^33^, and Src family kinases are not essential but are required for optimal uptake of IgG opsonised particles by macrophages^36^. Our data confirmed that efficient internalisation of ova ICs requires Src and Syk family kinase signalling (Supplementary Fig. S4). Despite this, however, PTPN22 (which dephosphorylates Src and Syk family kinases) was found to be dispensable for ova IC uptake (Fig. 3b). One likely explanation for this is that regulation of FcγR proximal kinase phosphorylation is mediated by phosphatases other than PTPN22. Tyrosine phosphatases including SHP-1 and SHP-2, in addition to inositol phosphatases SHIP-1 and SHIP-2 have each been demonstrated to regulate kinase signalling downstream of FcγRs. Another possibility is that PTPN22 exerts a subtle effect, fine tuning signals downstream of FcγRs, to specifically regulate MHCII antigen presentation, rather than broader BMDC effector functions. Our data does not allow us to distinguish if PTPN22 operates to control FcR dependent antigen presentation by regulating Eα_52–68_ peptide-MHCII formation, trafficking to the cell surface, or presentation of these complexes on the cell surface. Dendritic cells use endolysosomal tubules to transport peptide-MHCII complexes to the cell surface, for presentation to T cells^45^. In a murine B cell line, Syk was shown to be required for efficient formation of peptide-MHCII complexes^46^, and ITAM signalling is required to maintain peptide-MHCII complexes on the surface of dendritic cells^29^. These data may indicate that in the absence of PTPN22 mediated dephosphorylation, Syk activity is enhanced leading to increased formation of peptide-MHCII complexes on the cell surface of immune complex pulsed *Ptpn22*^*−/−*^ BMDCs.

Like PTPN22, fellow PTPN22 family member PTPN12^47^, is also dispensable for ova protein induced T cell proliferation, but is required for the induction of optimal IFNγ secretion by T cells^48^. These data indicate that members of the same phosphatase family can be redundant for antigen internalisation, yet act to regulate specific and independent DC effector functions. Indeed, since *Ptpn22*^*−/−*^ BMDCs will express PTPN12, this may also explain why IFNγ secretion was similar by T cells co-cultured with ova IC pulsed WT and *Ptpn22*^*−/−*^ BMDCs.

Since our data indicate that the absence of PTPN22 enhances FcγR dependent antigen presentation, it may have implications for individuals harbouring the *PTPN22*^*R620W*^ polymorphism. *PTPN22*^*R620W*^ affects the protein binding region of the P1 polyproline domain, and this mutation has been found to cause a gain- or loss-of-function depending on the cell type and signalling pathway being regulated. If PTPN22^R620W^ acts as a loss-of-function mutation in the context of FcγR signalling, then a lack of regulation of immune complex signalling in DCs could lead to enhanced T cell activation, augmenting immune responses to self-antigens. It would therefore be interesting in the future to assess the effect of PTPN22^R620W^ on FcγR dependent immune responses in dendritic cells, and the impact that this has on T cell activation.

## Materials and Methods

### Mice

Wild type (WT) C57BL/6, *Ptpn22*^*−/−*^ and OT-II mice were used in experiments as per protocols approved by the UK Home Office, and housed under specific pathogen free conditions. *Ptpn22*^*−/−*^ mice have been backcrossed for more than 10 generations to the C57BL/6 strain. Brownlie, R. J. *et al*.^49^ have previously described their generation and phenotype. All mice used in experiments were age and gender-matched.

### Bone marrow dendritic cell culture

BMDCs were produced using protocols adapted from Inaba, K. *et al*.^50^, and are described in detail in Clarke, F. *et al*.^30^.

### CD4^+^ T cell isolation

CD4^+^ T cells were isolated from lymph nodes and spleens of WT OT-II mice using CD4^+^ T cell negative isolation kits (Miltenyi Biotech). To monitor T cell proliferation, isolated T cells were resuspended at 2 × 10^7^/ml in PBS and labelled using CellTrace Violet (2 µM, CTV, Invitrogen) for 20 minutes at 37 °C, followed by quenching in culture media for 20 minutes at 37 °C.

### Ova immune complex production

The protocol for immune complex production is based on Ellsworth J *et al*.^51^. For immune complexes that will bind to all FcγRs, EndoGrade ovalbumin (ova, Hyglos GmbH) and rabbit anti-chicken egg albumin (anti-ova, Sigma) were combined at a ratio of 1:20 (ova:anti-ova) in PBS and incubated at 37 °C for 1 hour. Insoluble ova immune complexes (ova ICs) were pelleted at 17,200 g at 4 °C for 30 minutes and resuspended in PBS or culture media. Total protein concentration was determined by BCA assay (Pierce) as per the manufacturer’s instructions, and added to BMDCs at 1 µM. In some experiments, ICs were added at 10 µg/ml. ICs were stored at 4 °C and used within 1 week of production.

For isotype specific immune complexes, 2,4,6-Trinitrophenyl ovalbumin (TNP-ova, Santa Cruz Biotechnology) was combined with IgG1 specific anti-TNP (a kind gift from Dr. Michael Robson, King’s College London), and produced as above.

As additional controls for some experiments, BMDCs were also stimulated with 1 µM ova and 1 µM heat aggregated rabbit IgG (Sigma) or 1 µM ova pre-incubated with rabbit IgG (using the same procedure as making ova ICs, as above). Rabbit IgG was heat aggregated by being incubated at 62 °C for 20 minutes, cooled to 4 °C and centrifuged at 17,200 g to remove aggregates.

### DC-T cell co-cultures

WT and *Ptpn22*^*−/−*^ BMDCs were pulsed overnight with 1 µM ova or 1 µM ova ICs. The following day, BMDCs were harvested, washed and resuspended in DC culture media. BMDCs were seeded in 96 well round bottom plates and co-cultured for 1–6 days with CTV labelled WT OT-II CD4^+^ T cells at 1:2 (1 × 10^5^ BMDCs: 2 × 10^5^ T cells). After 1 day of co-culture, cell surface expression of CD25 and CD69 (clones PC61 and H1.2F3 respectively, BioLegend) was assessed by flow cytometry. After 3 and 6 days of co-culture, T cell proliferation (via CTV dilution) and intracellular cytokine expression was determined by flow cytometry, after 6 hours of restimulation with PMA (10 ng/ml), ionomycin (500 ng/ml) and monensin (BioLegend). Secretion of IL-2, IFNγ, TNFα and IL-17 was assessed in cell-free supernatants by immunoassay, as per the manufacturer’s instructions (BioLegend).

### Immune complex stimulation of BMDCs

To assess FcγR dependent BMDC maturation, ova ICs were added directly to culture wells on day 6–7 of culture; ova or rabbit anti-ova alone were used as controls. 24 hours later, BMDCs were harvested, washed, blocked with a non-fluorescent FcγRII/III antibody (clone 2.4G2, BioLegend) and stained for cell surface expression of CD54, CD80, CD86 and MHCII I-A^b^ (clones YN1/1.7.4, 16-10A1, GL-1 and AF6-120.1 respectively, BioLegend). To identify live DCs, cells were also stained with anti-CD11c (clone N418, BioLegend) and a viability marker (Zombie Fixable Viability dye, BioLegend). Cells were washed with FACS buffer (5% FBS, 1 mM EDTA in PBS) and fixed using 1% paraformaldehyde (PFA, Electron Microscopy Sciences) in PBS prior to flow cytometry. Maturation marker expression was determined by gating on live CD11c^+^ singlets.

To determine FcγR induced cytokine production, BMDCs were harvested on day 7 of culture, counted and replated in 96 well round bottom plates at 2 × 10^5^/well, and stimulated with ova, rabbit anti-ova, ova pre-incubated with rabbit IgG, or ova ICs for 3–24 hours, in the presence of polymyxin B (50 µg/ml, Sigma). Cell-free supernatants were removed and assessed for IL-6, TNFα, and IL-12/23p40 cytokine expression by immunoassay as per the manufacturer’s instructions (BioLegend).

### BMDC FcγR expression

For cell surface FcγR expression, BMDCs were harvested, washed and stained using the following antibodies: FcγRI-APC (clone X545/7.1, BioLegend), FcγRII/III-FITC (clone 2.4G2, BioLegend), FcγRIIb (clone K9.361 cl5; a kind gift from Prof. Jeffrey Ravetch, Rockefeller University) followed by goat anti-mouse AF488 (Invitrogen) and FcγRIV (clone MB1 9E9 cl27; also a kind gift from Prof. Jeffrey Ravetch) followed by goat anti-hamster FITC (AbD Serotec). FcγRIII expression was determined by using anti-FcγRIIb followed by FcγRII/III-FITC. Cells were washed with FACS buffer and fixed using 1% PFA in PBS prior to flow cytometry. FcγR expression was determined by gating on live CD11c^+^ singlets.

### Immune complex uptake, degradation and presentation assays

For immune complex uptake, BMDCs (2 × 10^5^) were incubated with ova-AF488 (10 µg/ml, Invitrogen) immune complexes on ice for 45 minutes. Cells were washed with cold FACS buffer and incubated at 37 °C for 0–60 minutes. Further immune complex uptake was prevented by returning the samples to ice and washing with cold PBS. Cells were stained using anti-CD11c (BioLegend), F(ab’)2 goat anti-rabbit IgG-AF647 (Invitrogen) and a viability marker (Zombie Fixable Viability dye, BioLegend). Cells were washed with FACS buffer and fixed using 1% PFA in PBS prior to flow cytometry. Cell surface ova-AF488 ICs were identified as F(ab’)2 goat anti-rabbit IgG-AF647^+^, as this would bind to rabbit anti-ova-containing ICs. BMDCs with internalised ova-AF488 ICs were identified by gating on F(ab’)2 goat anti-rabbit IgG-AF647^-^ live CD11c^+^ singlets. To identify the role of FcγRs and Src and Syk family kinases in immune complex uptake, WT BMDCs were pre-incubated with anti-FcγRI, II/III and IV for 15 minutes at 4 °C, Src inhibitor-1 (5 µM, Sigma) for 15 minutes at 37 °C, or Syk inhibitor II (5 µM, Calbiochem) for 15 minutes at 37 °C, prior to the ova-AF488 IC uptake assay (described above).

For immune complex degradation, 3 µm polystyrene beads (Polysciences) were coated overnight with ova-AF594 (0.5 mg/ml, Invitrogen) at 4 °C. The following day, ova coated beads were washed and incubated for 20 minutes on ice with rabbit anti-ova (50 μg/ml, Sigma). 1 × 10^5^ BMDCs and 2 × 10^6^ ova/anti-ova coated beads (1 DC:20 beads) were added to wells of a 96 well round bottom plate, centrifuged for 5 minutes at 750 g and incubated at 37 °C, 5% CO_2_ for 0–7 hours. To exclude non-internalised beads, BMDCs were washed and stained for 20 minutes on ice with F(ab’)2 goat anti-rabbit IgG-AF647 (Invitrogen). Cells were washed with FACS buffer and lysed for 10 minutes on ice in lysis buffer (0.5% NP-40, 50 mM Tris, 150 mM NaCl). Beads were washed with FACS buffer twice and transferred to FACS tubes for flow cytometry. Internalised beads were identified as F(ab’)2 goat anti-rabbit IgG-AF647^-^.

For immune complex derived antigen presentation, 2 × 10^5^ WT or *Ptpn22*^*−/−*^ BMDCs were seeded per well of a 96 well round bottom plate and incubated with immune complexes containing GFP-Eα (0.3 mg/ml, a kind gift from Erwan Boëdec, Paris 7 University) and anti-GFP (Invitrogen) for 5 or 18 hours at 37 °C, 5% CO_2_. BMDCs were harvested, washed and stained for 30 minutes on ice with anti-CD11c (BioLegend), anti-Eα_52–68_-biotin (eBioscience) and a viability marker (Fixable Viability Dye eFluor 506, eBioscience). Cells were washed with FACS buffer and stained at room temperature for 10 minutes with streptavidin-APC (BioLegend) in PBS. Cells were washed with FACS buffer and fixed using 1% PFA in PBS prior to flow cytometry. BMDCs were identified as live CD11c^+^ singlets. To measure cell surface MHCII expression, BMDCs in separate wells (which did not receive GFP-Eα:anti-GFP immune complexes) were also stained with anti-CD11c (BioLegend), anti-MHCII I-A^b^ (BioLegend) and a viability marker (Fixable Viability Dye eFluor 506, eBioscience).

### DC-T cell conjugate assay

BMDCs were incubated overnight with 1 μM ova or ova ICs. The following day, BMDCs were harvested, washed and resuspended in PBS at 1 × 10^7^/ml and labelled for 20 minutes at 37 °C using CellTrace Far Red (1 μM, CTFR, Invitrogen), followed by quenching in culture media for 20 minutes at 37 °C. CTFR labelled BMDCs and CTV labelled WT OT-II CD4^+^ T cells were added to 1.5 ml tubes at 1:2 (1 × 10^5^ BMDCs: 2 × 10^5^ T cells, in a total volume of 50 μl), centrifuged for 2 minutes at 25 g and incubated at 37 °C for 0–120 minutes. Cells were fixed using 3% PFA in PBS, transferred to FACS tubes and acquired by flow cytometry using a medium flow rate. Conjugates were identified as CTV^+^ CTFR^+^ events. To identify the role of Src and Syk family kinases in conjugate formation, WT BMDCs were pre-incubated with Src inhibitor-1 (5 µM, Sigma) or Syk inhibitor II (5 µM, Calbiochem) for 15 minutes at 37 °C, prior to the conjugate assay (described above).

### Data acquisition and analysis

Flow cytometry samples were acquired using a Fortessa II or FACSCanto II with FACS Diva software (all from BD). Data were analysed using Flowjo software version 10 (TreeStar). All graphs were plotted using Prism version 7 (GraphPad) and analysed for statistical significance using a 2-way ANOVA followed by Sidak’s or Tukey’s multiple comparisons test, Wilcoxon matched-pairs signed ranks test or a paired t-test. p < 0.05 was deemed statistically significant.

## Electronic supplementary material

Supplementary Figures

## Data Availability

All data generated or analysed during this study are included in the published article (and its Supplementary Information files).

## References

[1] Simkins HMA (2005). Association of the PTPN22 locus with rheumatoid arthritis in a New Zealand Caucasian cohort. Arthritis Rheum..

[2] Hinks A (2005). Association between the PTPN22 gene and rheumatoid arthritis and juvenile idiopathic arthritis in a UK population: Further support that PTPN22 is an autoimmunity gene. Arthritis Rheum..

[3] Plenge RM (2005). Replication of Putative Candidate-Gene Associations with Rheumatoid Arthritis in 14,000 Samples from North America and Sweden: Association of Susceptibility with PTPN22, CTLA4, and PADI4. Am. J. Hum. Genet.

[4] Begovich AB (2004). A missense single-nucleotide polymorphism in a gene encoding a protein tyrosine phosphatase (PTPN22) is associated with rheumatoid arthritis. Am J Hum Genet.

[5] Kyogoku C (2004). Genetic association of the R620W polymorphism of protein tyrosine phosphatase PTPN22 with human SLE. Am. J. Hum. Genet..

[6] Orozco G (2005). Association of a functional single-nucleotide polymorphism of PTPN22, encoding lymphoid protein phosphatase, with rheumatoid arthritis and systemic lupus erythematosus. Arthritis Rheum..

[7] Fedetz M (2006). The 1858T PTPN22 gene variant contributes to a genetic risk of type 1 diabetes in a Ukrainian population. Tissue Antigens.

[8] Bottini N (2004). A functional variant of lymphoid tyrosine phosphatase is associated with type I diabetes. Nat. Genet..

[9] Zheng W, She J-X (2005). Genetic Association Between a Lymphoid Tyrosine Phosphatase (PTPN22) and Type 1 Diabetes. Diabetes.

[10] Hasegawa K (2004). PEST domain-enriched tyrosine phosphatase (PEP) regulation of effector/memory T cells. Science.

[11] Dai X (2013). A disease-associated PTPN22 variant promotes systemic autoimmunity in murine models. J. Clin. Invest..

[12] Burn, G. L. *et al*. Superresolution imaging of the cytoplasmic phosphatase PTPN22 links integrin-mediated T cell adhesion with autoimmunity. *Sci. Signal*. **9** (2016). 10.1126/scisignal.aaf2195 27703032

[13] Wang Y (2013). The Autoimmunity-Associated Gene PTPN22 Potentiates Toll-like Receptor-Driven, Type 1 Interferon-Dependent Immunity. Immunity.

[14] Purvis HA (2018). Protein tyrosine phosphatase PTPN22 regulates IL-1β dependent Th17 responses by modulating dectin-1 signaling in mice. Eur. J. Immunol..

[15] Wang Y (2015). The Autoimmunity-Associated Gene PTPN22 Potentiates Toll-like Receptor-Driven, Type 1 Interferon-Dependent Immunity. Nat. Genet..

[16] Cloutier JF, Veillette A (1999). Cooperative inhibition of T-cell antigen receptor signaling by a complex between a kinase and a phosphatase. J. Exp. Med..

[17] Fiorillo E (2010). Autoimmune-associated PTPN22 R620W Variation Reduces Phosphorylation of Lymphoid Phosphatase on an Inhibitory Tyrosine Residue. J. Biol. Chem..

[18] Burn GL, Svensson L, Sanchez-Blanco C, Saini M, Cope AP (2011). Why is PTPN22 a good candidate susceptibility gene for autoimmune disease?. FEBS Lett..

[19] Zhang J (2011). The autoimmune disease–associated PTPN22 variant promotes calpain-mediated Lyp/Pep degradation associated with lymphocyte and dendritic cell hyperresponsiveness. Nat. Genet..

[20] Rieck M (2007). Genetic Variation in PTPN22 Corresponds to Altered Function of T and B Lymphocytes. J. Immunol..

[21] Fousteri G, Liossis S-NC, Battaglia M (2013). Roles of the protein tyrosine phosphatase PTPN22 in immunity and autoimmunity. Clin. Immunol..

[22] Bruhns P (2015). Properties of mouse and human IgG receptors and their contribution to disease models. Blood.

[23] Nimmerjahn F, Ravetch JV (2008). Fcγ receptors as regulators of immune responses. Nat. Immunol..

[24] Takai T (2002). Roles of Fc receptors in autoimmunity. Nat. Rev. Immunol..

[25] Kleinau BS, Martinsson P, Heyman B (2000). Induction and Suppression of Collagen-induced Arthritis Is Dependent on Distinct Fcy Receptors. J. Exp. Med..

[26] Maglione PJ, Xu J, Casadevall A, Chan J (2008). Fc Receptors Regulate Immune Activation and Susceptibility during Mycobacterium tuberculosis Infection. J. Immunol..

[27] de Haij S (2010). *In vivo* Cytotoxicity of Type I CD20 Antibodies Critically Depends on Fc Receptor ITAM Signaling. Cancer Res..

[28] Boross P (2014). FcR -Chain ITAM Signaling Is Critically Required for Cross-Presentation of Soluble Antibody-Antigen Complexes by Dendritic Cells. J. Immunol..

[29] Graham DB (2011). ITAM signaling in dendritic cells controls T helper cell priming by regulating MHC class II recycling ITAM signaling in dendritic cells controls T helper cell priming by regulating MHC class II recycling. Blood.

[30] Clarke, F. *et al*. Protein tyrosine phosphatase PTPN22 is dispensable for dendritic cell antigen processing and promotion of T-cell activation by dendritic cells. *PLoS One* **12** (2017). 10.1371/journal.pone.0186625 PMC5645108 29040339

[31] Rafiq K, Bergtold A, Clynes R (2002). Immune complex – mediated antigen presentation induces tumor immunity. J. Clin. Invest..

[32] van Montfoort N (2012). Fc Receptor IIb Strongly Regulates Fc Receptor-Facilitated T Cell Activation by Dendritic Cells. J. Immunol..

[33] Sedlik C (2003). A Critical Role for Syk Protein Tyrosine Kinase in Fc Receptor-Mediated Antigen Presentation and Induction of Dendritic Cell Maturation. J. Immunol..

[34] Woelbing F (2006). Uptake of *Leishmania major* by dendritic cells is mediated by Fcγ receptors and facilitates acquisition of protective immunity. J. Exp. Med..

[35] Guilliams M, Bruhns P, Saeys Y, Hammad H, Lambrecht BN (2014). The function of Fcγ receptors in dendritic cells and macrophages. Nat. Rev. Immunol..

[36] Fitzer-Attas CJ (2000). Fcgamma receptor-mediated phagocytosis in macrophages lacking the Src family tyrosine kinases Hck, Fgr, and Lyn. J. Exp. Med..

[37] Crowley MT (1997). A Critical Role for Syk in Signal Transduction and Phagocytosis Mediated by Fcγ Receptors on Macrophages. J. Exp. Med..

[38] Molfetta R (2014). Regulation of Fc receptor endocytic trafficking by ubiquitination. Front. Immunol..

[39] Li M (2017). The common, autoimmunity-predisposing 620Arg>Trp variant of PTPN22 modulates macrophage function and morphology. J. Autoimmun..

[40] Magarian Blander J, Medzhitov R (2006). Toll-dependent selection of microbial antigens for presentation by dendritic cells. Nature.

[41] Vermeren S (2016). PTPN22 Is a Critical Regulator of Fcγ Receptor–Mediated Neutrophil Activation. J. Immunol..

[42] Culshaw S, Millington OR, Brewer JM, McInnes IB (2008). Murine neutrophils present Class II restricted antigen. Immunol. Lett..

[43] Strzelecka A, Kwiatkowska K, Sobota A (1997). Tyrosine phosphorylation and Fcγ receptor-mediated phagocytosis. FEBS Lett..

[44] Kiefer F (1998). The Syk protein tyrosine kinase is essential for Fcgamma receptor signaling in macrophages and neutrophils. Mol. Cell. Biol..

[45] Vyas JM, Kim YM, Artavanis-Tsakonas K, Love JC, der V (2007). V. A. and P. H. Tubulation of class II MHC compartments is microtubule dependent and involves multiple endolysosomal membrane proteins in primary dendritic cells. J Immunol.

[46] Roux DL (2007). Syk-dependent Actin Dynamics Regulate Endocytic Trafficking and Processing of Antigens Internalized through the B-Cell Receptor. Mol. Biol. Cell.

[47] Rhee I, Veillette A (2012). Protein tyrosine phosphatases in lymphocyte activation and autoimmunity. Nat. Immunol..

[48] Rhee I, Davidson D, Souza CM, Vacher J, Veillette A (2013). Macrophage Fusion Is Controlled by the Cytoplasmic Protein Tyrosine Phosphatase PTP-PEST/PTPN12. Mol. Cell. Biol..

[49] Brownlie, R. J. *et al*. Lack of the Phosphatase PTPN22 Increases Adhesion of Murine Regulatory T Cells to Improve Their Immunosuppressive Function. *Sci. Signal* **5** (2012). 10.1126/scisignal.2003365 PMC5836999 23193160

[50] Inaba K (1992). Generation of Large Numbers of Dendritic Cells from Mouse Bone Marrow Cultures Supplemented with Granulocyte/Macrophage Colony-stimulating Factor. J Exp Med.

[51] Ellsworth JL (2007). Targeting Immune Complex-Mediated Hypersensitivity with Recombinant Soluble Human Fc RIA (CD64A). J. Immunol..

