# Supplementary material for: The protein tyrosine phosphatase PTPN22 negatively regulates presentation of immune complex derived antigens
Source: Sci Rep. 2018 Aug 23;8:12692. doi: 10.1038/s41598-018-31179-x (PMC6107551; doi:10.1038/s41598-018-31179-x)
Supplement: Supplementary file 1 — Supplementary Figures [file 41598_2018_31179_MOESM1_ESM.pdf]

Supplementary Figures for: The protein tyrosine phosphatase PTPN22 negatively regulates presentation of immune complex derived antigens

Authors: *Fiona Clarke[1], Harriet A Purvis[1], Cristina Sanchez-Blanco[1], Enrique Gutiérrez-Martinez[1], Georgina H Cornish[1], Rose Zamoyska[2], Pierre Guermonprez[1] and Andrew P Cope[1]

Address: [1]Centre for Inflammation Biology and Cancer Immunology, School of Immunology and Microbial Sciences, Faculty of Life Sciences and Medicine, King's College London, London, SE1 1UL, United Kingdom and [2]Institute of Immunology and Infection Research, Centre for Immunity, Infection and Evolution, University of Edinburgh, Edinburgh, EH9 3FL, United Kingdom.

*Corresponding author: Fiona Clarke, fiona.1.clarke@kcl.ac.uk

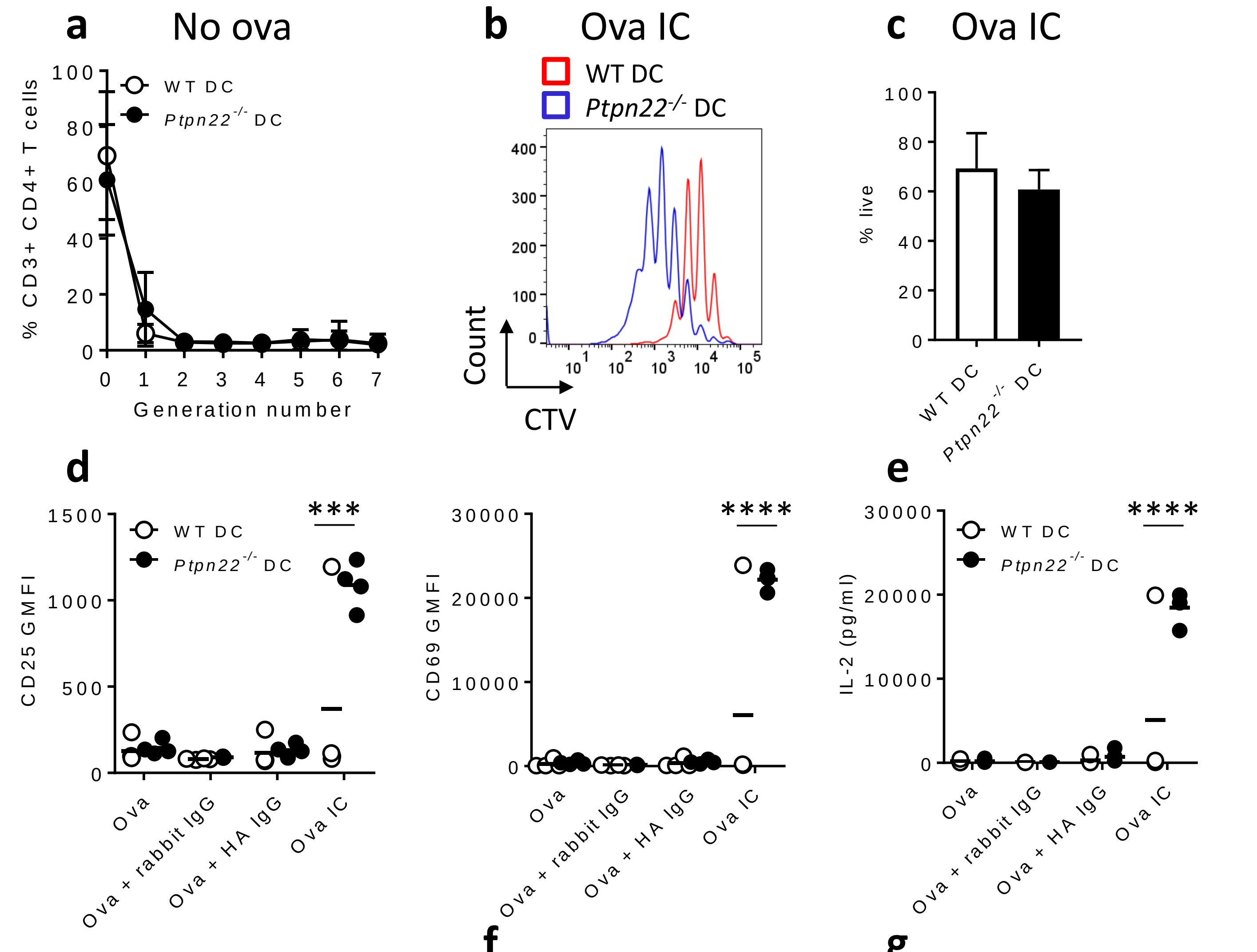

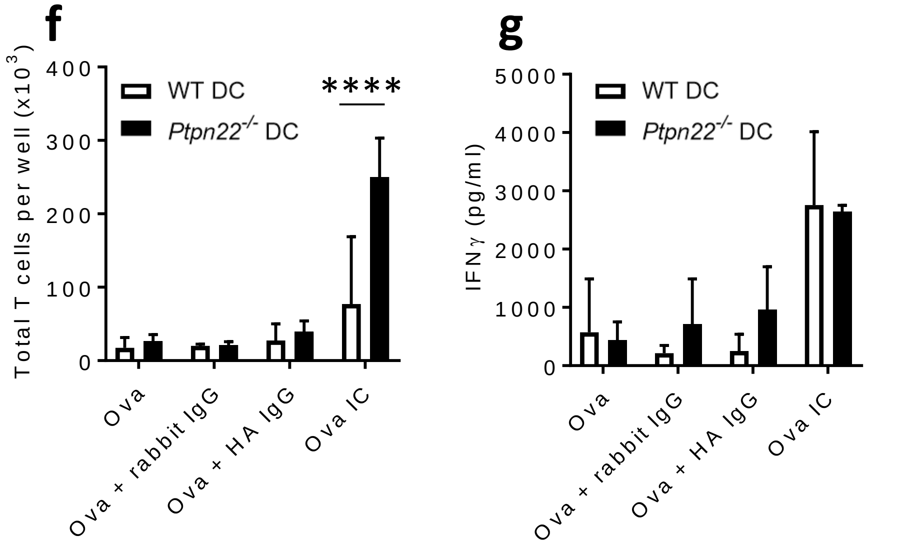

Supplementary Figure S1: Ova IC pulsed *Ptpn22-/-* BMDCs cause enhanced T cell activation and proliferation. (a) Proportion of WT OT-II T cells in each generation of cell division after 6 days of co-culture with unpulsed WT (white) or *Ptpn22-/-* (black) BMDCs (gated on live, CD3$^+$, CD4$^+$ singlets). n = 7-10 ± s.d. **(b)** Representative flow cytometry plot showing CTV dilution of WT OT-II T cells after 6 days of co-culture with WT (red) or *Ptpn22-/-* (blue) BMDCs. **(c)** WT OT-II T cell viability was assessed by flow cytometry using a live/dead viability marker, after 6 days of co-culture with ova IC pulsed WT (white) or *Ptpn22-/-* (black) BMDCs (gated on CD3$^+$, CD4$^+$ singlets). n = 7 + s.d. **(d-f)** WT or *Ptpn22-/-* BMDCs were pulsed overnight in the presence of ova, ova pre-incubated with rabbit IgG (ova + rabbit IgG), ova with heat aggregated IgG (ova + HA IgG), or ova IC prior to co-culture with CTV labelled WT CD4$^+$ OT-II T-cells for 1-3 days. **(d)** CD25 (left) and CD69 (right) cell surface expression (geometric mean fluorescence intensity, GMFI) on WT OT-II T cells after 1 day of co-culture with WT (white) or *Ptpn22-/-* (black) BMDCs (gated on live, CD3$^+$, CD4$^+$ singlets). n = 4; ****p<0.001, ***p<0.005 using a 2-way ANOVA with Sidak's multiple comparisons test. **(e)** At day 1 of co-culture, cell-free supernatants were assessed for IL-2 by immunoassay. n = 4; ****p<0.001 using a 2-way ANOVA with Sidak's multiple comparisons test. **(f)** The number of T cells per co-culture well was counted after 3 days of co-culture with WT (white) or *Ptpn22-/-* (black) BMDCs (gated on live, CD3$^+$, CD4$^+$ singlets). n = 4 + s.d; ****p<0.001 using a 2-way ANOVA with Sidak's multiple comparisons test. **(g)** At day 3 of co-culture, cell-free supernatants were assessed for IFNγ by immunoassay. n = 4 + s.d.

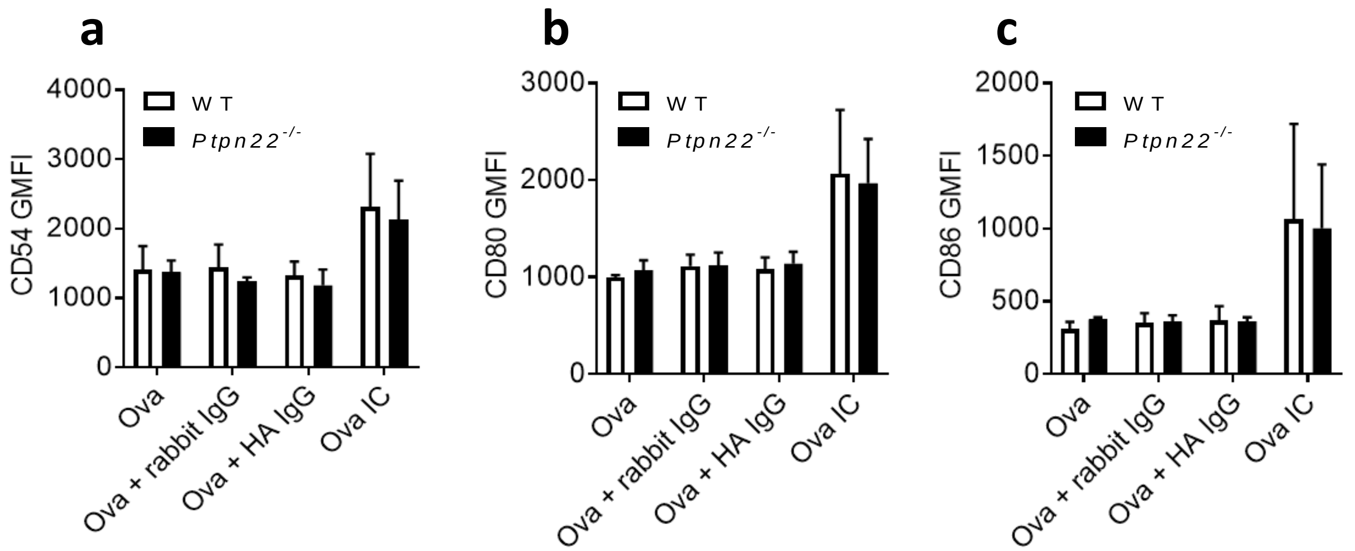

Supplementary Figure S2: PTPN22 is dispensable for upregulation of co-stimulatory molecules after immune complex stimulation. **(a-c)** WT or *Ptpn22*$^{-/-}$ BMDCs were stimulated in the presence of ova, ova pre-incubated with rabbit IgG (ova + rabbit IgG), ova with heat aggregated IgG (ova + HA IgG), or ova IC. After 18 hours, BMDCs were harvested and surface stained for CD54, CD80 and CD86. Pooled data (geometric mean fluorescence intensity, GMFI, gated on live, CD11c$^+$ singlets) are shown for WT (white) and *Ptpn22*$^{-/-}$ (black) BMDC CD54 **(a)**, CD80 **(b)** and CD86 **(c)** expression. n = 4 + s.d.

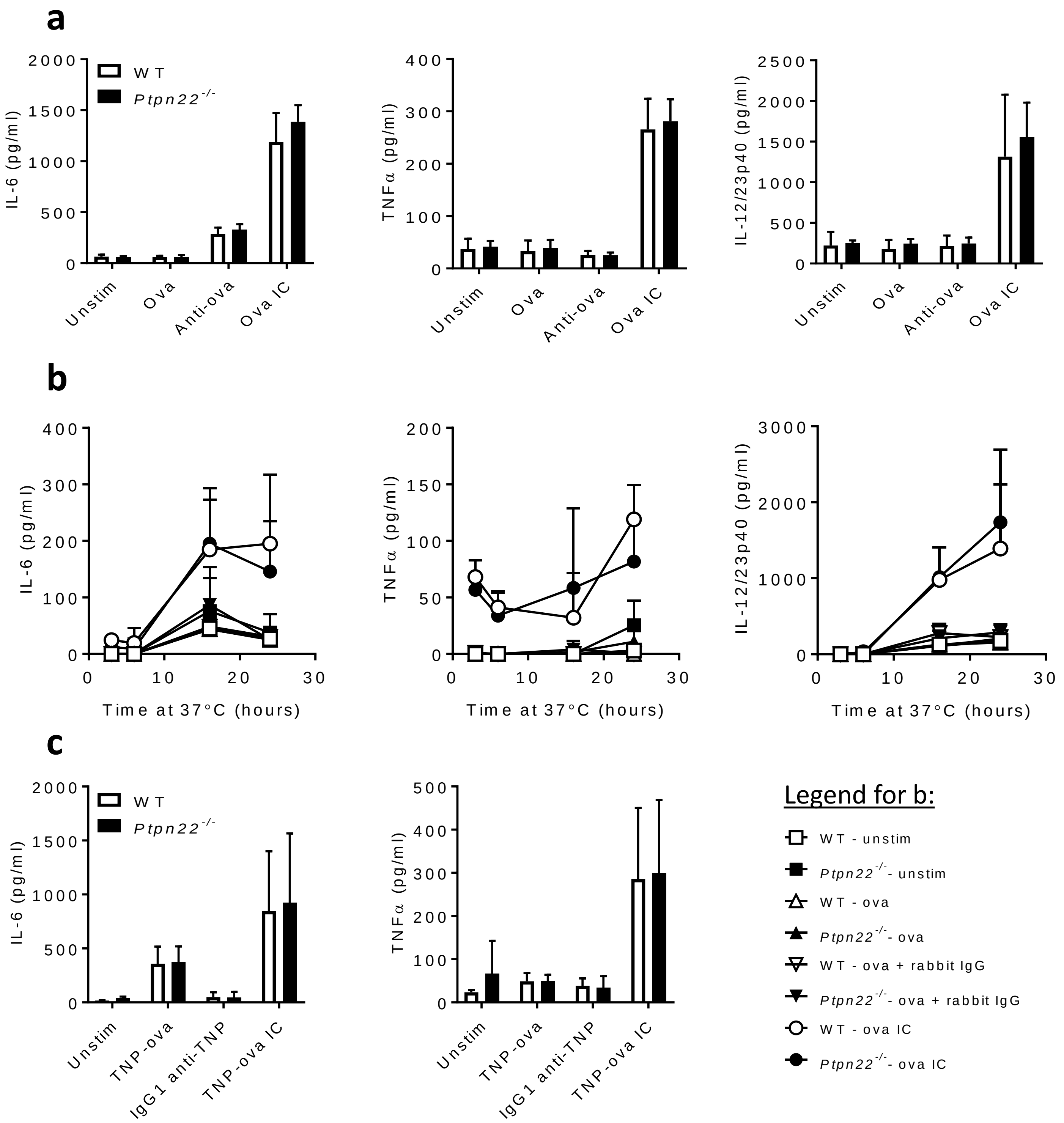

Supplementary Figure S3: PTPN22 is dispensable for BMDC cytokine secretion after immune complex stimulation. **(a)** WT (white) or *Ptpn22*$^{-/-}$ (black) BMDCs were stimulated in the presence of ova, anti-ova or ova IC. After 24 hours, cell-free supernatants were harvested and secretion of IL-6 (left), TNFα (middle) and IL-12/23p40 (right) was determined by immunoassay. n = 3 + s.d. (representative of 10 independent experiments). **(b)** WT (white) or *Ptpn22*$^{-/-}$ (black) BMDCs were stimulated in the presence ova (triangle), ova pre-incubated with rabbit IgG (ova + rabbit IgG, upside down triangle), ova IC (circle), or left unstimulated (unstim, square). After 3-24 hours, cell-free supernatants were harvested and secretion of IL-6 (left), TNFα (middle) and IL-12/23p40 (right) was determined by immunoassay. n = 4 + s.d. **(c)** WT (white) or *Ptpn22*$^{-/-}$ (black) BMDCs were stimulated in the presence of TNP-ova, IgG1 anti-TNP or TNP-ova IC. After 24 hours, cell-free supernatants were harvested and secretion of IL-6 (left) and TNFα (right) was determined by immunoassay. n = 3 + s.d.

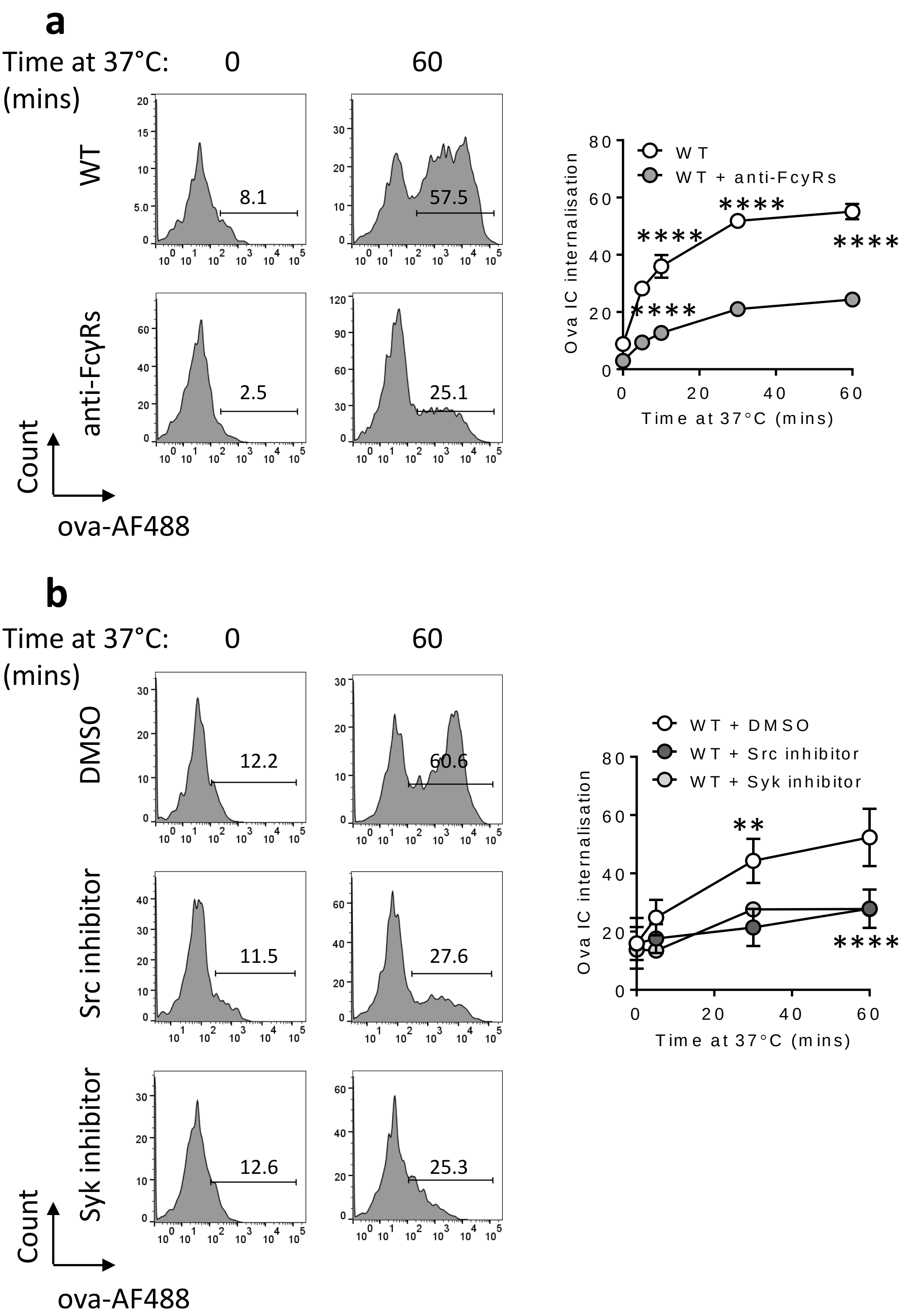

Supplementary Figure S4: FcγR mediated immune complex uptake is dependent on Src and Syk family kinases. (a) Representative flow cytometry plots (left) and combined data (right) of WT BMDC ova-AF488 IC internalisation with (grey) or without (white) pre-incubation with anti-FcγRs (%, gated on live CD11c$^+$, anti-rabbit$^-$ singlets). n = 3 ± s.d.; ****p<0.0001 using 2-way ANOVA with Sidak's multiple comparisons test. **(b)** Representative flow cytometry plots (left) and combined data (right) of WT BMDC ova-AF488 IC internalisation after pre-treatment with DMSO (vehicle control, white), Src inhibitor (dark grey) or Syk inhibitor (grey) (%, gated on live CD11c$^+$, anti-rabbit$^-$ singlets). n = 7, 4, 3 respectively ± s.d.; **p<0.01, ****p<0.0001 using 2-way ANOVA with Tukey's multiple comparisons test.

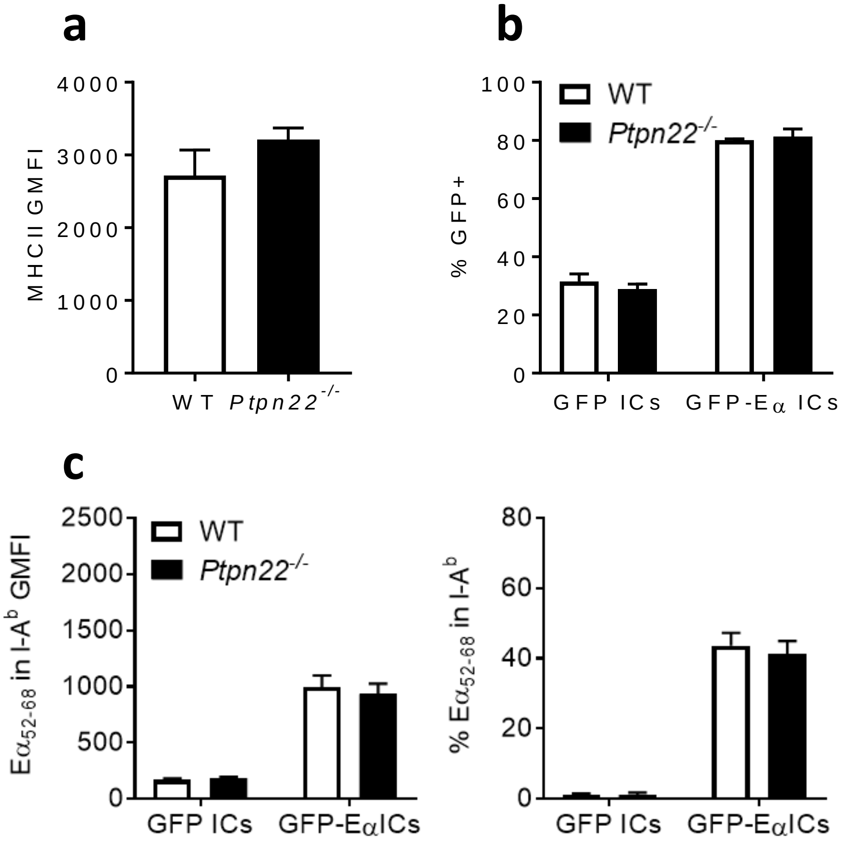

Supplementary Figure S5: PTPN22 is dispensable for immune complex uptake, and presentation of immune complex derived antigens is similar between WT and *Ptpn22$^{-/-}$* BMDCs at an earlier timepoint. (a) MHCII expression on WT (white) and *Ptpn22$^{-/-}$* (black) BMDCs (gated on live, CD11c^{+} singlets). n = 3 + s.d. **(b)** GFP:anti-GFP IC and GFP-Eα:anti-GFP IC uptake after 18 hours by WT (white) and *Ptpn22$^{-/-}$* (black) BMDCs (% GFP^{+}, gated on live, CD11c^{+} singlets). n = 3 + s.d. **(c)** Geometric mean fluorescence intensity (GMFI, left) and % positive (right) of $E\alpha_{52\text{-}68}$ surface expression in I-A^{b} by WT (white) and *Ptpn22$^{-/-}$* (black) BMDCs after 5 hour incubation with GFP/anti-GFP ICs and GFP-Eα/anti-GFP ICs (gated on live, CD11c^{+} singlets). n = 3 + s.d.

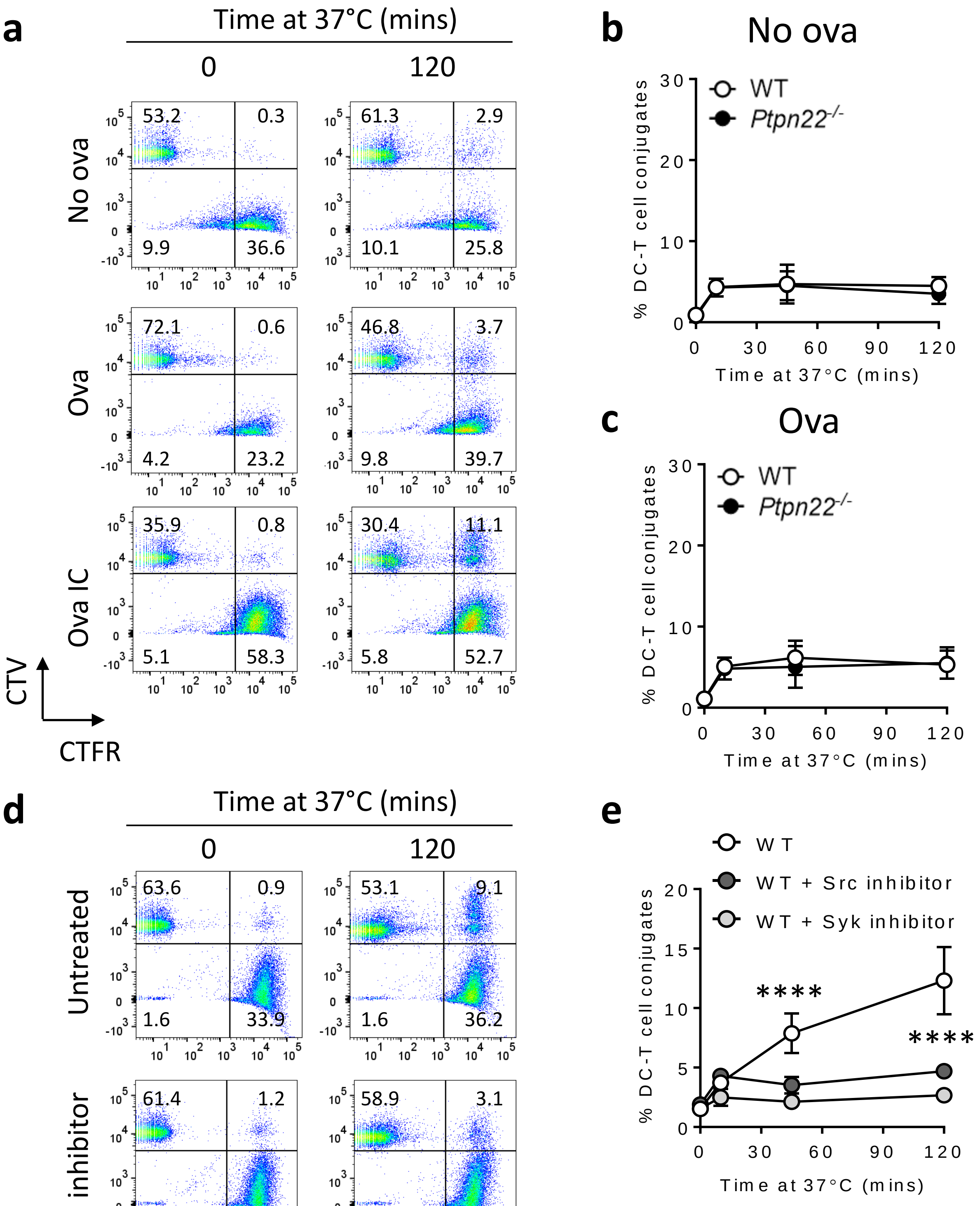

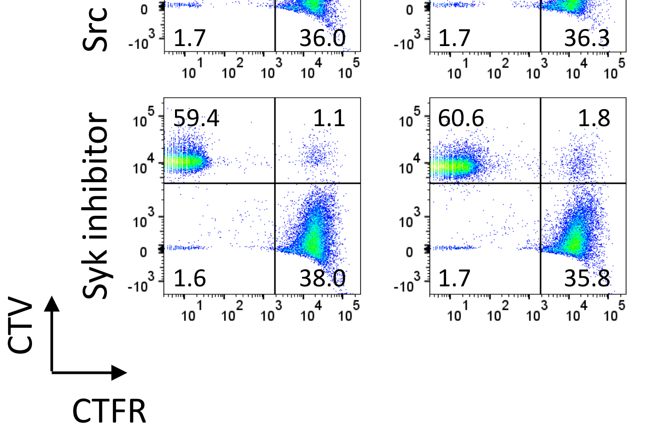

Supplementary Figure S6: Formation of DC-T cell conjugates by antigen pulsed BMDCs. (a-c) Representative flow cytometry plots **(a)** and combined data **(b and c)** of DC-T cell conjugates with WT (white) and *Ptpn22*$^{-/-}$ (black) BMDCs pulsed with ova, ova IC, or left unpulsed (no ova). n = 4 ± s.d. **(d-e)** Representative flow cytometry plots **(d)** and combined data **(e)** for the proportion of WT OT-II T cells in conjugates with ova IC pulsed WT BMDCs, after pre-treatment with Src inhibitor (dark grey), Syk inhibitor (grey), or with no pre-treatment (white). Conjugates were identified as CTV$^+$ CTFR$^+$ events (%, gated on CTV$^+$ total T cells). n = 3 ± s.d.; ****p<0.0001 using a 2-way ANOVA with Tukey's multiple comparisons test.

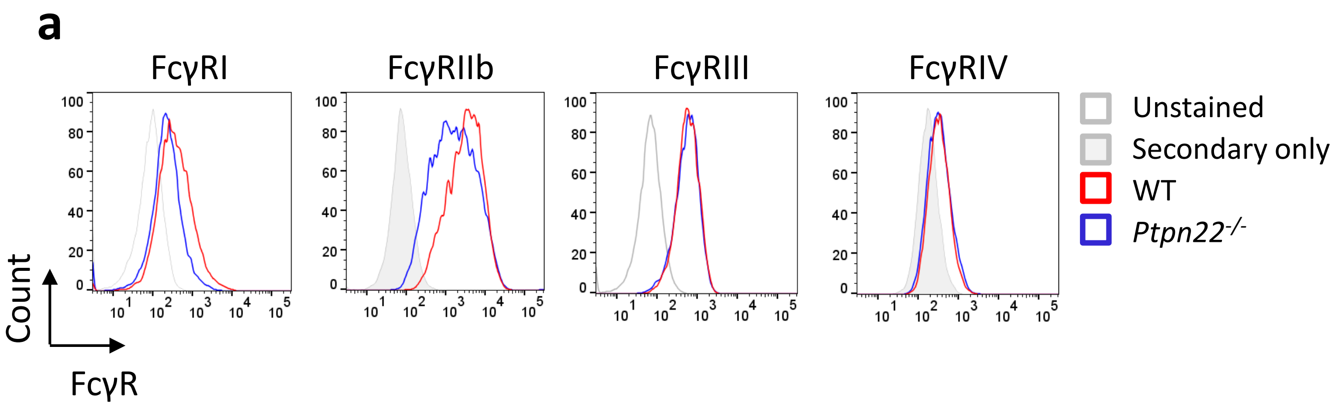

Supplementary Figure S7: PTPN22 regulates FcγRIIb expression on BMDCs. (a) Representative flow cytometry plots showing cell surface FcγR expression on WT (red) and *Ptpn22*$^{-/-}$ (blue) BMDCs (gated on live, CD11c^{+} singlets).
